# Artificial intelligence in cancer research: learning at different levels of data granularity

**DOI:** 10.1002/1878-0261.12920

**Published:** 2021-02-20

**Authors:** Davide Cirillo, Iker Núñez‐Carpintero, Alfonso Valencia

**Affiliations:** ^1^ Barcelona Supercomputing Center (BSC) Barcelona Spain; ^2^ ICREA Barcelona Spain

**Keywords:** artificial intelligence, cancer research, data granularity, machine learning

## Abstract

From genome‐scale experimental studies to imaging data, behavioral footprints, and longitudinal healthcare records, the convergence of big data in cancer research and the advances in Artificial Intelligence (AI) is paving the way to develop a systems view of cancer. Nevertheless, this biomedical area is largely characterized by the co‐existence of big data and small data resources, highlighting the need for a deeper investigation about the crosstalk between different levels of data granularity, including varied sample sizes, labels, data types, and other data descriptors. This review introduces the current challenges, limitations, and solutions of AI in the heterogeneous landscape of data granularity in cancer research. Such a variety of cancer molecular and clinical data calls for advancing the interoperability among AI approaches, with particular emphasis on the synergy between discriminative and generative models that we discuss in this work with several examples of techniques and applications.

## Graphical abstract

Cancer research is largely characterized by the co‐existence of big data and small data with varied sample sizes, label availability, and data types. The interplay among distinct artificial intelligence approaches allows learning at different levels of data granularity, having the potential to improve prevention and diagnosis, patient stratification, and treatment decisions.

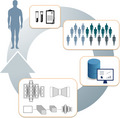

**Abbreviations**
AI: Artificial Intelligence
CEDCD: Cancer Epidemiology Descriptive Cohort Database
EGA: European Genome‐phenome Archive
EHR: Electronic Health Record
FDA: Food and Drug Administration
GAN: Generative Adversarial Network
HLA: Human Leukocyte Antigen
HPC: High Performance Computing
MHC: Major Histocompatibility Complex
TCGA: The Cancer Genome Atlas

## 1 Introduction

Data granularity refers to the level of detail observable in the data. The finer the granularity, the more detailed are the observations. In cancer research, data granularity reflects the amount of molecular and clinical information that is collected about a patient or a group of patients, not only in terms of dataset size but also in terms of diversity of measurements, scales, and data types. At present, the available data in cancer research may not always provide the level of granularity required for effective decision‐making. For instance, healthcare resources exhibit a shortage of information about specific cancer subtypes, minority groups, and rare cancers, such as the case of pediatric oncology [1]; national cancer registries tend to collect mainly first‐line treatments and display reduced accessibility to actionable information [2]; and exigent legal and ethical approvals hurdle the timeliness of cancer data availability [3]. In this scenario, several initiatives devoted to some of these facets have been created, such as the Collaboration for Oncology Data in Europe (CODE; www.code‐cancer.com), Rare Cancers Europe (RCE; www.rarecancerseurope.org), and the Cancer Drug Development Forum (CDDF) [4]. Nevertheless, the granularity of oncological data is highly scattered worldwide, resulting in a continuum of scale, quality, and completeness of the available datasets, that we refer to as *data continuum*. This aspect is particularly relevant in the context of the development of Artificial Intelligence (AI) systems, which are largely characterized by data‐intensive computational modeling approaches to assist clinical decision‐making [5, 6, 7].

**Fig. 1:**
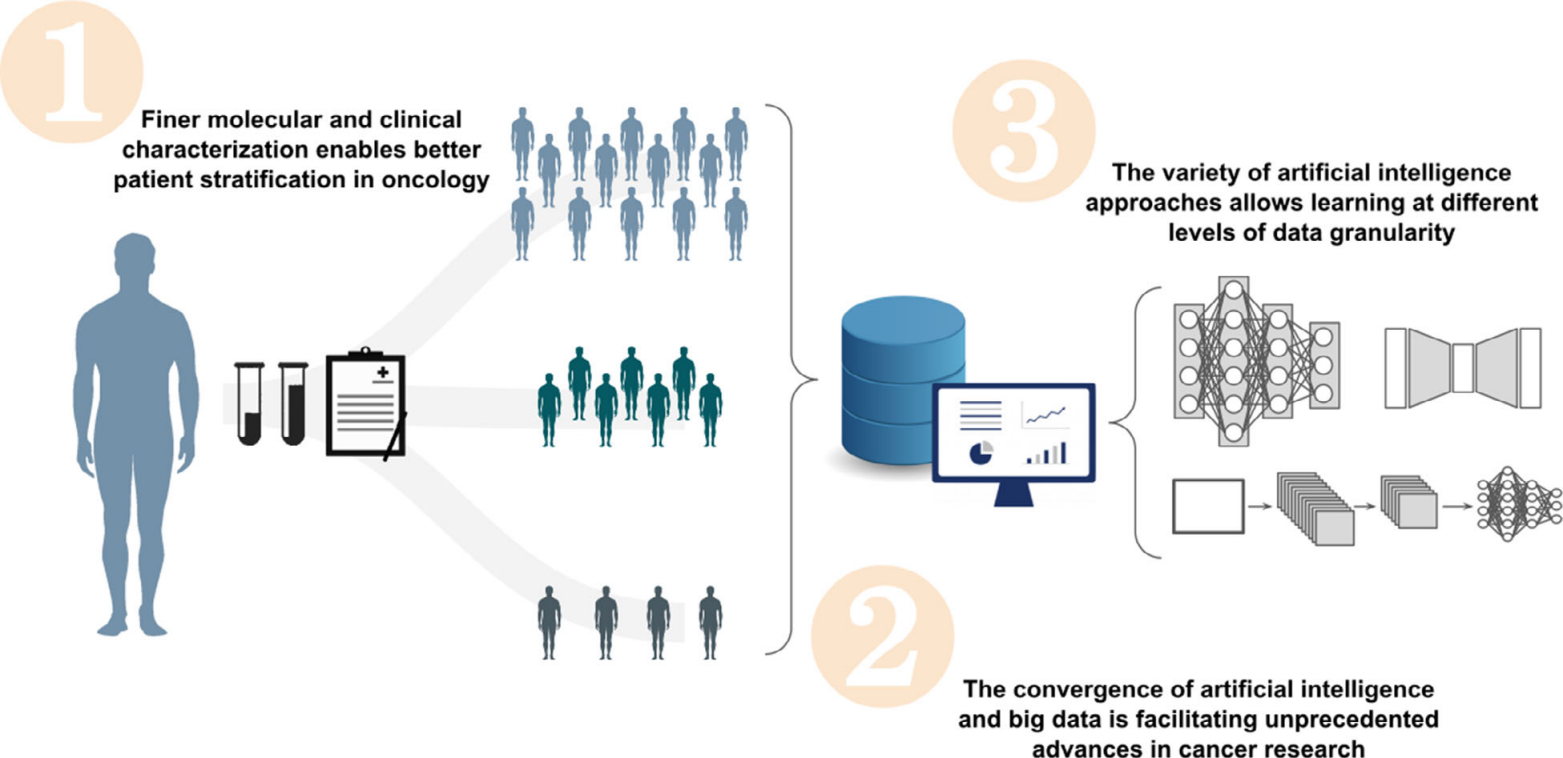
The interplay between data generated with different levels of granularity and the multiplicity of AI approaches in cancer research.

In this work, we examine how cancer data granularity (from population studies to subgroups stratification) relates to multiple AI approaches (from deep learning to linear regression), and provide possible solutions to reconcile the interoperability between these two components to ensure modeling strategies within the data continuum (Fig. 1). This work brings forward the specific need of developing AI techniques able to transcend the current limitations in their applications to the heterogeneous levels of granularity typical of cancer datasets.

The article is structured in three parts. In the first part, we analyze the ongoing process of confluence of big data and AI in cancer research (‘Big data in cancer research’ and ‘The role of AI in cancer research’), and report on the main data types and areas of application (‘Main areas of application and data types of AI in cancer research’). In the second part, we challenge the current focus on big data by examining two large‐scale projects, namely the Cancer Genome Atlas (TCGA) and the Cancer Epidemiology Descriptive Cohort Database (CEDCD), under the lens of data granularity (‘Heterogeneous levels of data granularity in cancer research’), and provide an overview on multiple AI approaches that allow learning at different levels of data granularity as well as discuss challenges and limitations (‘Sample size and label availability: limitations and solutions’). In the third part, we deliver the conclusions to the article and a perspective view on the future of AI in cancer research (‘Conclusions and Perspectives’).

## 2 Big data in cancer research

Cancer research has been witnessing unprecedented innovations in recent years, including a major paradigm shift from histological level to molecular level characterization of cancers with a strong impact on treatment and medical practice [8, 9]. An illustrative example of this change is the current, finer categorization of blood cancers into multiple subtypes based on the patient's genetic information [10]. Moreover, new technologies, such as CRISPR gene editing [11] and CAR T‐cell therapy [12], are pushing the frontiers of clinical intervention and research. Additionally, single‐cell multi‐omics and imaging of preclinical personalized cancer models, such as organoids [13], are proving extremely valuable in dissecting key aspects of tumor evolution, as demonstrated by the research activities of initiatives such as LifeTime [14].

Such variety of data, including structure and unstructured clinical and molecular information (e.g., genetic tests, medical records, imaging data), outlines a horizon of possibilities for advancing oncology. Efforts to fill the gap between molecular and clinical information have been proposed, such as the concept of the Patient Dossier [15], which aims to facilitate the information flow between complex genomic pipelines and basic queries involving several aspects of the patient's health. Nevertheless, the progress in our understanding of cancer is not dependent on the sole availability of large amounts of high‐quality and diversified data. The ongoing accumulation of records on a large number of patients is reinforcing the pressing need of cancer research and clinical care to embrace computational solutions to effectively utilize all this information. The effective utilization of cancer big data entails all the steps from data processing and storage to data mining, analysis, and final applications, such as the identification of patient‐specific oncogenic processes [16] and biomarkers [17]. Moreover, the continuous improvement of data quality through standardization procedures that ensure responsible molecular and clinical data sharing, interoperability, and security is a key aspect for cancer research that is strongly catalyzed by initiatives such as the Global Alliance for Genomics and Health (GA4GH; https://www.ga4gh.org).

As traditional data management methods cannot handle the scale and variety of cancer data acquired and generated daily, advanced infrastructures for permanent archiving and sharing are presently flourishing. An example of an extensive repository of data resulting from biomedical research projects is the European Genome‐phenome Archive (EGA; https://ega‐archive.org/). EGA collects various data types, including public access data (e.g., somatic mutation, gene expression, anonymized clinical data, protein expression) and controlled access data (e.g., germline genetic variants). EGA stores data from cancer‐centric data sources, including TCGA, the International Cancer Genome Consortium (ICGC), the Clinical Proteomic Tumor Analysis Consortium (CPTAC), and the OncoArray Consortium.

## 3 The role of AI in cancer research

Although advanced solutions for big data management are facilitating the handling of biomedical information, the road to clinical success (e.g., better prevention and diagnosis, improved treatment decisions, effective patient‐clinical trial matching) must involve ways to leverage the data and to be able to gain actionable insights from it [18, 19]. Predictive analytics and machine learning are thriving areas of research and application in cancer research, characterized by interdisciplinarity and diversity of approaches, which henceforth we collectively refer to as AI. At present, 6 Food and Drug Administration (FDA)‐approved AI‐based radiological devices with applications in oncology are available for mammography analyses and computer tomography (CT)‐based lesion detection [20], and 74 AI algorithms for digital pathology have received FDA clearance [21]. Moreover, more than 300 AI‐related clinical trials have been registered at ClinicalTrial.gov [22] and seven randomized trials assessing AI in medicine have been published [23]. These examples are some of the many AI systems that stem from research and development advances in real‐time decision‐making for health care, which are systematically surveyed and compared [24].

Biomedical big data coupled with the ability of machines to learn and find solutions to problems have ensured that AI is currently playing a major role in the progress of biomedicine [25, 26, 27] and particularly cancer research [28, 29]. Indeed, big data and AI complement each other, as AI feeds off of big data, from which it can learn how to carry out tasks such as classifying groups of patients, forecasting disease progression, and delivering adaptive treatment recommendations. AI and big data have the potential to fathom and overcome issues such as the reliability of biomarkers and genetic information [30, 31], the potential disparities in patient populations [32, 33], and the limited understanding of side effects [34] despite the growing promise of combination therapy [35, 36] and drug repurposing [37].

The convergence of AI and big data can help interlace the threads of the complex landscape of oncological medicine resources, which is currently pervaded by a high level of heterogeneity and lack of standards [38]. In this regard, international efforts, such as the European‐Canadian Cancer Network (EUCANCan; https://eucancan.com/) and individualizedPaediatricCure (https://ipc‐project.eu/), are advancing the potential of federated data infrastructures to improve standardized data reporting and the development of cancer‐specific AI solutions.

To facilitate this progress, automated strategies for end‐to‐end AI processes operating on big data, from data governance to deployment of AI applications, have been developed. The intensive workloads of AI operating on big data demand computational resources that must be able to achieve extreme scale and high performance while being cost‐effective and environmentally sustainable [39]. High performance computing (HPC), or supercomputing, architectures are facilitating the deployment of pioneering AI applications in biomedicine [40, 41]. In this view, HPC represents a critical capacity to gain competitive advantages, including not only faster and more complex computation schemes but also at lower costs and higher impact. Innovative software and hardware solutions, as well as model training implementations that support fine‐grained parallelism and restrain memory costs, aim to accelerate the forthcoming convergence of AI and HPC. For this reason, community‐driven benchmarking infrastructures for objective and quantitative evaluation of bioinformatics methods and algorithms [42, 43] as well as domain‐specific evaluation campaigns [44] are acquiring an increasing importance within the cancer research community.

## 4 Main areas of application and data types of AI in cancer research

The variety of modalities of available data (i.e., molecular profiles, images, texts) enables the full potential of AI in cancer research. For instance, imaging data has been used to train AI models for skin cancer classification [45] and lymph node metastasis detection [46], while sequencing data has been used for variant functional impact assessment [47] and patient survival prediction [48]. These examples employ artificial neural networks, specifically deep learning, which has marked the biggest trend in AI over the last decade [49]. Deep learning has largely been applied to cancer data integration and modeling, such as the classification of medical images and digital health data, often in combination with processing of electronic health records (EHRs), and included in systems supporting physician–computer interactions [50].

In an ideal scenario, a comprehensive collection of cancer patient data should include both data derived from the patient (e.g., demographic information, familial history, symptoms, comorbidities, histopathological features, immunohistochemistry, nucleic acid sequencing, biochemical analyses, digital images, experience measurements using digital devices) but also results generated from the application of AI. In this regard, the main AI implementations in cancer research encompass (a) statistical and mathematical models of the system under study and (b) simulations of such models aiming to explore the system's properties and behavior in different conditions. The main data types employed in such models and simulations comprise multi‐omics and immunogenomics data, longitudinal data (e.g., EHRs), behavioral data (e.g., wearable devices and social media), and imaging data [51].

Multi‐omics data play a central role in cancer research. Given the interplay between different biological phenomena (e.g., gene expression, epigenetic modifications, protein–protein interactions), the development of approaches to integrate multiple layers of data has become a subject of profound interest in this area. Harmonizing such heterogeneous sources of information represents a challenge that, in recent years, has led to the development of platforms that leverage data of large‐scale pan‐cancer initiatives and offer analytical functions, such as LinkedOmics [52] and DriverDBv3 [53].

Recent developments in AI for cancer research are contributing significantly to the field of cancer immunology, in particular neoantigen prediction. Thanks to the predictive power of deep learning, large‐scale sequencing data of neoantigens and major histocompatibility complex (MHC) molecules can be used to test possible binding of truncated proteins of a tumor cell and the patient's human leukocyte antigen (HLA) system, enabling the discovery of treatment targets that would be both patient‐ and tumor‐specific. Following this concept, a recent study was able to validate a personalized vaccine for melanoma using candidate neoantigens obtained with a tool using deep learning, NetMHCpan [54, 55]. Other recently developed tools using deep learning are devoted to the prediction of antigen presentation in the context of HLA‐class II, such as MARIA [56] and NetMHCIIpan [57]. Being promising targets for personalized immunotherapies, neoantigen prediction is a blooming area for which expert recommendations have been recently set out by the European Society for Medical Oncology (ESMO) including optimal selection schemes for candidate prioritization, pipelines for binding affinity prediction and mutated peptide annotation and comparison [58].

Deep learning is widely employed in the processing and analysis of medical imaging data which has resulted in a wide variety of applications, achieving remarkable results in prognosis prediction from routinely obtained tissue slides [59], tumor detection and classification [45, 60] and, more recently, real‐time tumor diagnosis [61, 62].

It is important to note that the collection of EHRs is growing at levels comparable to those of genomic and molecular data. In this regard, EHRs represent a type of data whose processing has proven AI particularly challenging. Indeed, the high variety of clinical terminology, highly specialized words, abbreviations and short notes, makes EHRs content processing through general‐purpose Natural Language Processing (NLP) models extremely arduous. Recent efforts focus on the generation of unified semantic systems and the organization of community challenges [63] from which automatically annotated corpora can be derived, which will facilitate the progress in this area [64, 65]. One of the main challenges that all these advanced technologies, including modern approaches to digital and systems medicine, are currently facing is their integration and clinical exploitation in the health systems [66]. Indeed, many complex aspects, such as regulation, commercialization, and ethics, are playing a central role in the operational transformation of modern cancer care. For instance, despite the astounding advances in smartphones and Internet of Things (IoT) technologies, which largely facilitate the collection of patient‐generated health data, regulatory priorities and positions as well as limitations in device‐based data analytics directly affect the slow uptake of such digital medicine solutions in oncology [67].

## 5 Heterogeneous levels of data granularity in cancer research

Despite the availability of cancer big data, a prominent feature of the current data landscape in oncology is the imbalance between the amount of data per patient and the cohort size. Indeed, while thousands to millions observables per patient are routinely generated, a typical cohort size of specific groups of patients is relatively small [68].

**Fig. 2:**
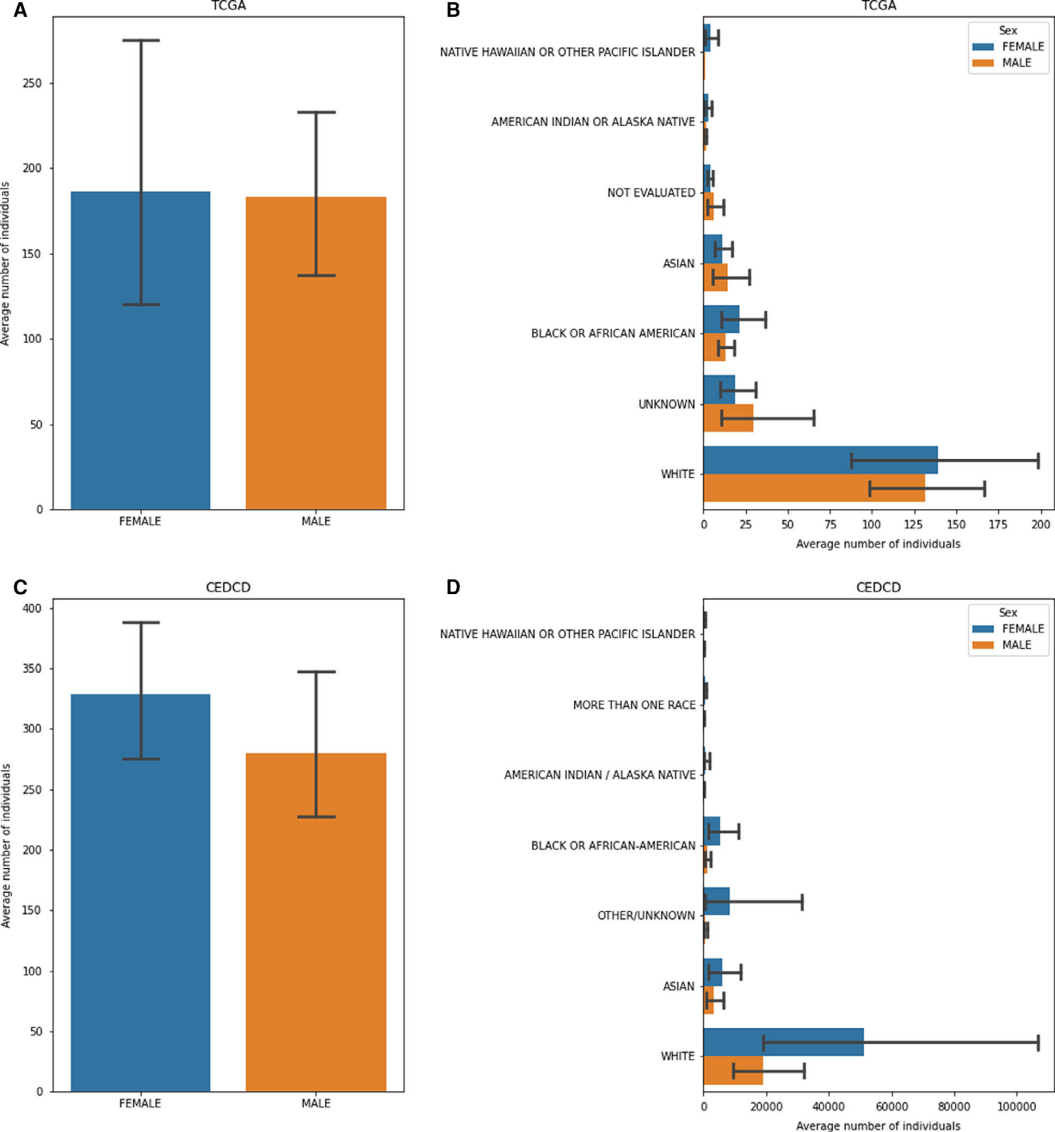
Demographic features of the individuals represented in TCGA and CEDCD projects. (A) Average number of individuals per cancer type in TCGA disaggregated by sex; (B) average number of individuals per cancer type in TCGA disaggregated by race and sex; (C) average number of individuals per cancer type in CEDCD cohort studies disaggregated by sex; and (D) average number of individuals per CEDCD cohort studies disaggregated by race and sex.

As an example, we examine the curated clinical data of TCGA project [69] (Fig. 2A,B). The average number of unique patients per cancer type (*N* = 33) is 335.78 (on average, 182.93 male and 186.32 female individuals). As expected, these numbers reduce when disaggregated by race (on average, 131.62 White, 13.43 Black or African American, 14.64 Asian male individuals, and 139.12 White, 21.17 Black or African American, 10.96 Asian female individuals) and, even more, by tumor stage, if this annotation is available. For instance, the 87 patients with mesothelioma, a rare but fatal cancer causally linked to asbestos exposure [70], distribute unevenly in the six stages (stage I, IA, IB, II, III, IV) by sex and race. White males are the most represented patients (80.4%), mostly appearing in late stages, reflecting both the gradual onset of the disease [71] and its incidence in developing countries that have consumed asbestos over past decades (83% in males and 17% in females as of 2017 in the United Kingdom; source: https://www.cancerresearchuk.org/). This observation highlights not only the overriding importance of early detection and better risk assessment tools based on socio‐economic factors but also the need for effective AI‐based approaches to learn from the little data that might be available.

A similar trend can be observed in prospective cohort studies, such as those reported in the CEDCD (https://cedcd.nci.nih.gov/), which collects large observational population studies aimed to prospectively investigate the environmental, lifestyle, clinical, and genetic determinants of cancer incidence (Fig. 2C,D). As of September 2020, the average number of participants diagnosed with cancer per cohort (*N* = 61) is 14624.65. However, when disaggregated by sex and cancer type (*N* = 25), this number decreases to an average of 328.50 women and 279.75 men per cancer type in each cohort. Also, the cohort composition is markedly skewed toward specific race categories, with an average of 19 172.77 White, 1330.83 Black or African American, 3420.39 Asian male participants, and 51 347.72 White, 5446.14 Black or African American, 6058.08 Asian female participants per cohort. These observations highlight the need for devising better strategies to improve the low enrollment rates in cohort studies and overcome the obstacles to minority populations engagement [72, 73].

## 6 Sample size and label availability: limitations and solutions

In the area of cancer research, a long‐standing challenge is the insufficient availability of massive high‐quality labeled datasets coupling exhaustive molecular profiles with matching detailed clinical annotations [18]. In the current scattered scenario, there is a growing need to exploit the multiplicity of AI approaches for the nonexclusive utilization of the available data with different levels of granularity.

Most AI applications in cancer research are mainly based on two types of learning algorithms: supervised and unsupervised learning [74, 75, 76]. Supervised learning involves models that map data instances to labels in order to perform tasks such as classification and regression. Unsupervised learning involves models that extract information from data instances without labels to perform tasks such as clustering and dimensionality reduction. Additionally, many hybrid types of learning (e.g., semi‐supervised learning) as well as specific learning techniques (e.g., transfer learning) are largely employed. All these approaches can be either discriminative or generative, whether they estimate the conditional probability of a label given an instance or the conditional probability of an instance given a label, respectively [77]. Thus, discriminative models can distinguish between different instances, while generative models can produce new ones.

**Fig. 3:**
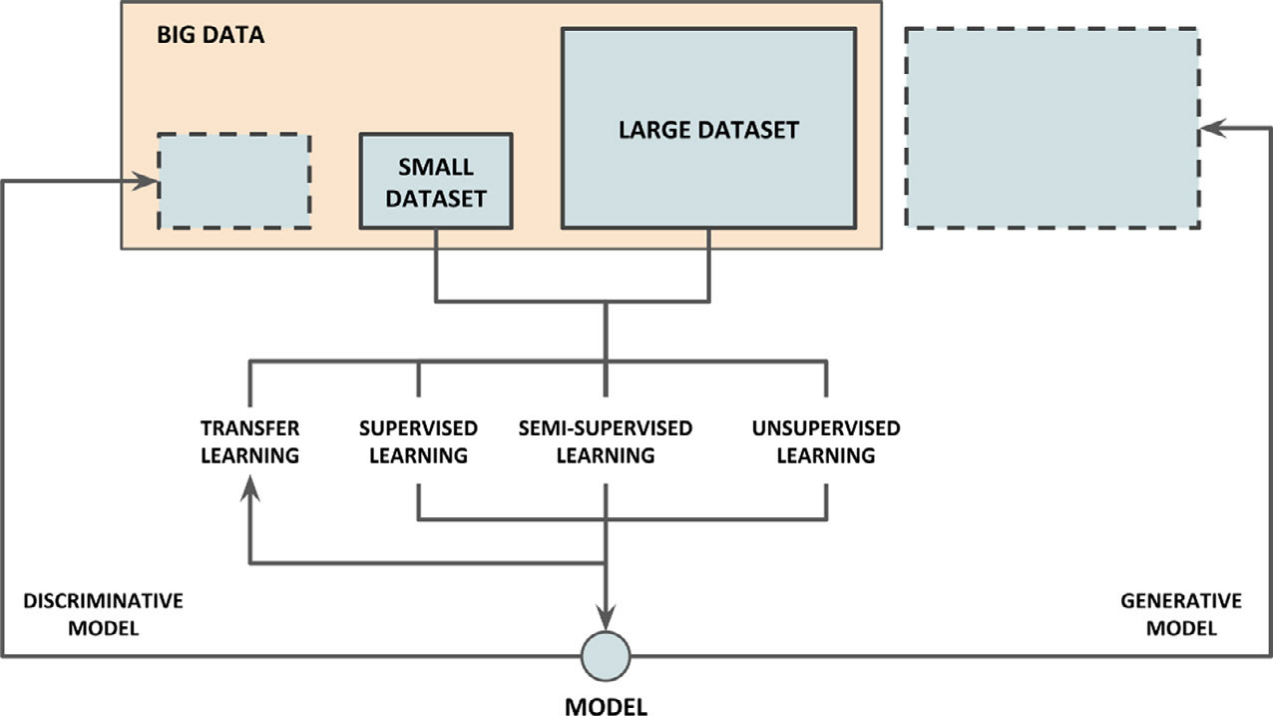
Synergy of AI solutions for cancer research in the data continuum. Based on label availability of large and small datasets (e.g., over‐ and under‐represented cancer subgroups), several learning approaches (supervised, semi‐supervised, unsupervised, transfer learning) can be attained to create both generative and discriminative models. While discriminative models can be used to identify smaller subsets from the totality of big data (represented as small dashed rectangle on the upper left corner), generative models can be used for data augmentation by producing large volumes of synthetic instances (represented as a large dashed rectangle on the upper right corner).

Label availability and the varied scales of cancer data call for advancing the interoperability among AI approaches, in particular the synergy of discriminative and generative models. These models can be used, in turn, for inference and data augmentation, feeding back a finer characterization and accessibility of data for further training (Fig. 3).

Label availability can guide the choice of an AI approach or another for either discriminative or generative purposes. The dearth of ground‐truth labels which are necessary to perform supervised tasks represents one of the main limitations to the use of AI in many areas of cancer research. The collection, curation and validation of labels by experts is an expensive and laborious process resulting in datasets that are too small to estimate complex models required to answer complex questions [78]. Models with low statistical power may lead to nonconvergence as well as biased and inadmissible outcomes, undermining reproducibility and reliability. Beside limited label availability and sample size, other limiting factors for AI can be identified, such as number of features, depth of hyperparameter optimization, and number of cross‐validation folds [79].

When informative and defensible background information is available (e.g., previous studies, meta‐analyses, expert knowledge), Bayesian statistics may produce reasonable results with small sample sizes [80, 81, 82]. Indeed, well‐considered decisions are strongly endorsed in the choice of ‘thoughtful’ priors as opposed to naïvely using Bayesian estimation in small sample contexts. Nevertheless, prior information about the distribution of the parameters cannot be explicitly available and often difficult to derive.

If only a very limited amount of labels is available, AI approaches operating with minimal training data exist, including transfer learning and meta‐learning techniques for few‐, one‐, and zero‐shot learning (surveyed in [83, 84]). As an example, re‐using a model trained on high‐resource language pairs, such as French‐English, can improve translation on low‐resource language pairs, such as Uzbek‐English [85]. Due to the ability of learning from minimal data, transfer learning and meta‐learning are increasingly gaining momentum having the potential to mitigate many criticisms over deep learning concerning the requisite extensive computational resources and training data [86].

Transfer learning re‐uses the weights of pretrained models in a similar learning task [87]. For instance, it has been recently applied to model anticancer drug response in a small dataset transferring the information learnt from large datasets [88]. This study illustrates the potential of transfer learning to improve future drug response prediction performance on patients by transferring information from patient‐derived models, such as xenografts and organoids. Nevertheless, although transfer learning is designed to transfer information from a support domain to a target domain, very limited target training data can hamper the efficient adaptation to a new task even with shared features between the support and target data.

Meta‐learning is based on the concept of ‘learning to learn’ consisting of improving performance over multiple learning episodes instead of multiple data instances. Meta‐learning learns from the meta‐data of previously experienced tasks, including model configurations (e.g., hyperparameter settings), evaluations (e.g., accuracies), and other measurable properties, enabling the search of an optimal model, or combinations of models, for a new task [89]. Recently, meta‐learning has been applied to the prediction of cancer survival [90]. Despite the high adaptability of meta‐learning, this study shows how the related tasks used for training should contain a reasonable amount of transferable information to achieve a significant improvement in performance compared to other learning strategies. For instance, if the samples of a specific cancer display very unique and distinct features, learning directly from them may represent a more effective strategy than learning from other cancer samples.

If the training data are only partially labeled, semi‐supervised learning techniques, such as pseudolabeling and entropy minimization, proved successful and, for this reason, dedicated standard evaluation practices have been recently devised [91]. Semi‐supervised learning jointly uses unlabeled and a smaller set of labeled data to improve the performances of one or both unsupervised and supervised tasks using the information learnt from the other or both [92]. Inherent limitations of semi‐supervised learning mainly include strong assumptions about the feature space carrying relevant information about the prediction task. In this regard, the assumed dependency between labeled and unlabeled sets is deemed to effectively reveal fitting decision boundaries for predictive models. However, it has been shown that causal tasks, such as semantic segmentation in cancer imaging analysis, do not comply with these assumptions [93] and high‐quality supervised baselines are crucial to assess the added value of unlabeled data in semi‐supervised learning settings.

If enough labeled data are initially available for training, data augmentation can be achieved using generative models based on neural networks, such as generative adversarial networks (GANs) [94], variational autoencoders [95], and transformer models [96]. These approaches display technical open challenges that need further investigation, for instance the training instability and low mode diversity of GANs [97]. Oversampling datasets can also be achieved by creating synthetic instances to increase the training data and avoid class imbalance [98]. Moreover, similar to image data augmentation techniques and synonym replacement in texts, other methods based on data manipulations and new instances interpolation, such as the Synthetic Minority Oversampling Technique (SMOTE) algorithm [99], have been proposed.

Synthetic data generation represents a promising solution to the ethical and privacy barriers that may prevent in‐depth data analysis and modeling of patients' information. For instance, the generation of synthetic data points has been exploited as a privacy‐preserving approach to overcome the limitations and difficulties of data anonymization [100]. Indeed, instead of partially de‐identifying data or censoring and removing protected variables, synthetic patient records can be fabricated from real‐world data and used for model development and healthcare applications testing. Moreover, synthetic data can also be generated to specifically mirror the clinical features of a patient, thus creating a so‐called digital twin or avatar for computationally evaluation of personalized drug treatments [101].

## 7 Conclusions and perspectives

Cancer is a disease that exhibits features of complex systems (e.g., self‐organization, emerging patterns, adaptive and collective behavior, nonlinear dynamics). Cancer complexity is exemplified by the definition of the so‐called hallmarks of cancer [102], which holds a systems view of the disease to be investigated through computational approaches. Computational cancer research is a multidisciplinary area aimed to advance the biomedical understanding of cancer by harnessing the power of data analytics and AI to advance in both basic and clinical settings [103, 104]. With the rapid development of precision medicine and big data applications in cancer research, AI is setting down exceptional opportunities and ambitious challenges in this area [105, 106], facilitating the progress toward individually tailored preventive and therapeutic interventions. The acquisition of a deep understanding of such interindividual differences relies on the development of AI systems that enable the identification of biomedically relevant patterns from several data from multiple modalities, spanning a varied range of data types, and displaying heterogeneous levels of granularity. Among the many details defining data granularity in cancer research, such as scales, measurements, and data types, sample size and label availability are the most evident factors that have a direct impact on the application of AI in cancer research. The range of AI modeling approaches that allow learning from both large and small datasets to discriminate or generate observations show the extraordinary potential of operating within a continuum of dataset sizes. This synergy among multiple learning techniques, namely supervised, semisupervised, transfer, and unsupervised learning, encompasses the entire spectrum of data granularity, including both the effective generalization from few examples with applications to multidimensional data, and the effective ability of models trained on big data to uncover small subgroups and subtle details. These AI approaches are not short of limitations and general assumptions that need to be considered before naïvely apply them. In this regard, it is particularly important to develop robust systems for testing and benchmarking AI applications, with adequate data resources and cleaver strategies that can be converted into certifications for the use of AI in real‐world medical scenarios, as recently proposed for diagnostic imaging algorithms [107]. We envisage a growing use of such a multiplicity of AI approaches in cancer research that will enable an interconnected integration of automatic learning processes within the data continuum, from big data to small data as well as from small data to big data.

## Conflict of interest

The authors declare no conflict of interest.

## Author contributions

AV and DC conceived the study. All the authors, AV, DC, and IN‐C, contributed to the writing of the article.

## Data Availability

The code to reproduce the barplots in Fig. 2 is available at: https://github.com/cirillodavide/cancer_data_granularity.

## References

[1] Pui CH , Gajjar AJ , Kane JR , Qaddoumi IA & Pappo AS (2011) Challenging issues in pediatric oncology. Nat Rev Clin Oncol 8, 540–549. 21709698 10.1038/nrclinonc.2011.95 PMC3234106

[2] Pop B , Fetica B , Blaga ML , Trifa AP , Achimas‐Cadariu P , Vlad CI & Achimas‐Cadariu A (2019) The role of medical registries, potential applications and limitations. Med Pharm Rep 92, 7–14. 30957080 10.15386/cjmed-1015 PMC6448488

[3] Mascalzoni D , Dove ES , Rubinstein Y , Dawkins HJS , Kole A , McCormack P , Woods S , Riess O , Schaefer F , Lochmüller H *et al*. (2015) International charter of principles for sharing bio‐specimens and data. Eur J Hum Genet 23, 721–728. 25248399 10.1038/ejhg.2014.197 PMC4795058

[4] Verweij J , Hendriks HR & Zwierzina H (2019) Cancer drug development forum innovation in oncology clinical trial design. Cancer Treat Rev 74, 15–20. 30665053 10.1016/j.ctrv.2019.01.001

[5] van der Ploeg T , Austin PC & Steyerberg EW (2014) Modern modelling techniques are data hungry: a simulation study for predicting dichotomous endpoints. BMC Med Res Methodol 14, 137. 25532820 10.1186/1471-2288-14-137 PMC4289553

[6] Steyerberg EW , Uno H , Ioannidis JPA & van Calster B (2018) Collaborators poor performance of clinical prediction models: the harm of commonly applied methods. J Clin Epidemiol 98, 133–143. 29174118 10.1016/j.jclinepi.2017.11.013

[7] Richter AN & Khoshgoftaar TM (2018) A review of statistical and machine learning methods for modeling cancer risk using structured clinical data. Artif Intell Med 90, 1–14. 30017512 10.1016/j.artmed.2018.06.002

[8] Ogino S , Fuchs CS & Giovannucci E (2012) How many molecular subtypes? Implications of the unique tumor principle in personalized medicine. Expert Rev Mol Diagn 12, 621–628. 22845482 10.1586/erm.12.46 PMC3492839

[9] Loomans‐Kropp HA & Umar A (2019) Cancer prevention and screening: the next step in the era of precision medicine. NPJ Precis Oncol 3, 3. 30701196 10.1038/s41698-018-0075-9 PMC6349901

[10] Taylor J , Xiao W & Abdel‐Wahab O (2017) Diagnosis and classification of hematologic malignancies on the basis of genetics. Blood 130, 410–423. 28600336 10.1182/blood-2017-02-734541 PMC5533199

[11] Stadtmauer EA , Fraietta JA , Davis MM , Cohen AD , Weber KL , Lancaster E , Mangan PA , Kulikovskaya I , Gupta M , Chen F *et al*. (2020) CRISPR‐engineered T cells in patients with refractory cancer. Science 367, 6481. 10.1126/science.aba7365 PMC11249135 32029687

[12] Mohseni YR , Tung SL , Dudreuilh C , Lechler RI , Fruhwirth GO & Lombardi G (2020) The future of regulatory T cell therapy: promises and challenges of implementing CAR technology. Front Immunol 11, 1608. 32793236 10.3389/fimmu.2020.01608 PMC7393941

[13] Kim J , Koo BK & Knoblich JA (2020) Human organoids: model systems for human biology and medicine. Nat Rev Mol Cell Biol 21, 571–584. 32636524 10.1038/s41580-020-0259-3 PMC7339799

[14] Rajewsky N , Almouzni G , Gorski SA , Aerts S , Amit I , Bertero MG , Bock C , Bredenoord AL , Cavalli G , Chiocca S *et al*. (2020) LifeTime and improving European healthcare through cell‐based interceptive medicine. Nature 587, 377–386. 32894860 10.1038/s41586-020-2715-9 PMC7656507

[15] Vazquez M & Valencia A (2019) Patient dossier: healthcare queries over distributed resources. PLoS Comput Biol 15, e1007291. 31622330 10.1371/journal.pcbi.1007291 PMC6797086

[16] Vasudevan S , Flashner‐Abramson E , Remacle F , Levine RD & Kravchenko‐Balasha N (2018) Personalized disease signatures through information‐theoretic compaction of big cancer data. Proc Natl Acad Sci USA 115, 7694–7699. 29976841 10.1073/pnas.1804214115 PMC6065026

[17] Crichton DJ , Altinok A , Amos CI , Anton K , Cinquini L , Colbert M , Feng Z , Goel A , Kelly S , Kincaid H *et al*. (2020) Cancer biomarkers and big data: a planetary science approach. Cancer Cell 38, 757–760. 32976775 10.1016/j.ccell.2020.09.006

[18] Azuaje F (2019) Artificial intelligence for precision oncology: beyond patient stratification. NPJ Precis Oncol 3, 6. 30820462 10.1038/s41698-019-0078-1 PMC6389974

[19] Clarke MA & Fisher J (2020) Executable cancer models: successes and challenges. Nat Rev Cancer 20, 343–354. 32341552 10.1038/s41568-020-0258-x

[20] Benjamens S , Dhunnoo P & Meskó B (2020) The state of artificial intelligence‐based FDA‐approved medical devices and algorithms: an online database. NPJ Digit Med 3, 509. 10.1038/s41746-020-00324-0 PMC7486909 32984550

[21] ACR Data Science Institute FDA Cleared AI Algorithms. https://www.acrdsi.org/DSI‐Services/FDA‐Cleared‐AI‐Algorithms

[22] CONSORT‐AI and SPIRIT‐AI Steering Group (2019) Reporting guidelines for clinical trials evaluating artificial intelligence interventions are needed. Nat Med 25, 1467–1468. 31551578 10.1038/s41591-019-0603-3

[23] Topol EJ (2020) Welcoming new guidelines for AI clinical research. Nat Med 26, 1318–1320. 32908274 10.1038/s41591-020-1042-x

[24] Ahmed Z & Mohamed K (2000) Artificial intelligence with multi‐functional machine learning platform development for better healthcare and precision medicine. Database 2000, baa010. 10.1093/database/baaa010 PMC7078068 32185396

[25] Agrawal R & Prabakaran S (2020) Big data in digital healthcare: lessons learnt and recommendations for general practice. Heredity 124, 525–534. 32139886 10.1038/s41437-020-0303-2 PMC7080757

[26] Singh O , Singh R & Saxena A (2020) AI and precision medicine for oncology. Proceedings of the International Conference on Innovative Computing & Communications (ICICC). 10.2139/ssrn.3566788.

[27] Goecks J , Jalili V , Heiser LM & Gray JW (2020) How machine learning will transform biomedicine. Cell 181, 92–101. 32243801 10.1016/j.cell.2020.03.022 PMC7141410

[28] Ho D (2020) Artificial intelligence in cancer therapy. Science 367, 982–983. 32108102 10.1126/science.aaz3023

[29] Liang G , Fan W , Luo H & Zhu X (2020) The emerging roles of artificial intelligence in cancer drug development and precision therapy. Biomed Pharmacother 128, 110255. 32446113 10.1016/j.biopha.2020.110255

[30] Shi W , Ng CKY , Lim RS , Jiang T , Kumar S , Li X , Wali VB , Piscuoglio S , Gerstein MB , Chagpar AB *et al*. (2018) Reliability of whole‐exome sequencing for assessing intratumor genetic heterogeneity. Cell Rep 25, 1446–1457. 30404001 10.1016/j.celrep.2018.10.046 PMC6261536

[31] Reiter JG , Baretti M , Gerold JM , Makohon‐Moore AP , Daud A , Iacobuzio‐Donahue CA , Azad NS , Kinzler KW , Nowak MA & Vogelstein B (2019) An analysis of genetic heterogeneity in untreated cancers. Nat Rev Cancer 19, 639–650. 31455892 10.1038/s41568-019-0185-x PMC6816333

[32] Zavala VA , Bracci PM , Carethers JM , Carvajal‐Carmona L , Coggins NB , Cruz‐Correa MR , Davis M , de Smith AJ , Dutil J , Figueiredo JC *et al*. (2021) Cancer health disparities in racial/ethnic minorities in the United States. Br J Cancer 124, 315–332. 32901135 10.1038/s41416-020-01038-6 PMC7852513

[33] Li CH , Prokopec SD , Sun RX , Yousif F & Schmitz N (2020) PCAWG tumour subtypes and clinical translation, boutros, P.C., PCAWG Consortium Sex differences in oncogenic mutational processes. Nat Commun 11, 4330. 32859912 10.1038/s41467-020-17359-2 PMC7455744

[34] Grassberger C , Ellsworth SG , Wilks MQ , Keane FK & Loeffler JS (2019) Assessing the interactions between radiotherapy and antitumour immunity. Nat Rev Clin Oncol 16, 729–745. 31243334 10.1038/s41571-019-0238-9 PMC13492067

[35] Zervantonakis I (2020) Improving cancer combination therapy by timing drug administration. Sci Transl Med 12, eabb5671.

[36] Bayat Mokhtari R , Homayouni TS , Baluch N , Morgatskaya E , Kumar S , Das B & Yeger H (2017) Combination therapy in combating cancer. Oncotarget 8, 38022–38043. 28410237 10.18632/oncotarget.16723 PMC5514969

[37] Zhang Z , Zhou L , Xie N , Nice EC , Zhang T , Cui Y & Huang C (2020) Overcoming cancer therapeutic bottleneck by drug repurposing. Signal Transduct Target Ther 5, 113. 32616710 10.1038/s41392-020-00213-8 PMC7331117

[38] Montouchet C , Thomas M , Anderson J & Foster S (2018) The oncology data landscape in Europe: Report. European Federation of Pharmaceutical Industries and Associations. https://www.efpia.eu/media/412192/efpia‐onco‐data‐landscape‐1‐report.pdf

[39] Strubell E , Ganesh A & McCallum A . (2020) Energy and policy considerations for modern deep learning research. In Proceedings of the The Thirty‐Fourth AAAI Conference on Artificial Intelligence, AAAI 2020, The Thirty‐Second Innovative Applications of Artificial Intelligence Conference, IAAI 2020, The Tenth AAAI Symposium on Educational Advances in Artificial Intelligence, EAAI 2020, New York, NY, USA, February 7‐12, 2020, AAAI Press, pp. 13693–13696.

[40] Kovatch P , Gai L , Cho HM , Fluder E & Jiang D (2020) Optimizing high‐performance computing systems for biomedical workloads. In Proceedings of the 2020 IEEE International Parallel and Distributed Processing Symposium Workshops (IPDPSW), pp. 183–192. 10.1109/ipdpsw50202.2020.00040 PMC7575271 33088611

[41] Castrignanò T , Gioiosa S , Flati T , Cestari M , Picardi E , Chiara M , Fratelli M , Amente S , Cirilli M , Tangaro MA *et al*. (2020) ELIXIR‐IT HPC@CINECA: high performance computing resources for the bioinformatics community. BMC Bioinformatics 21, 333. 32838759 10.1186/s12859-020-03565-8 PMC7446135

[42] Capella‐Gutierrez S , de la Iglesia D , Haas J , Lourenco A , Fernández JM , Repchevsky D , Dessimoz C , Schwede T , Notredame C , Gelpi JL *et al*. (2017) Lessons learned: recommendations for establishing critical periodic scientific benchmarking. bioRxiv 181677 [PREPRINT]. 10.1101/181677.

[43] Rappoport N & Shamir R (2018) Multi‐omic and multi‐view clustering algorithms: review and cancer benchmark. Nucleic Acids Res 46, 10546–10562. 30295871 10.1093/nar/gky889 PMC6237755

[44] Hirschman L , Yeh A , Blaschke C & Valencia A (2005) Overview of BioCreAtIvE: critical assessment of information extraction for biology. BMC Bioinformatics 6 (Suppl 1), S1. 10.1186/1471-2105-6-S1-S1 PMC1869002 15960821

[45] Esteva A , Kuprel B , Novoa RA , Ko J , Swetter SM , Blau HM & Thrun S (2017) Dermatologist‐level classification of skin cancer with deep neural networks. Nature 542, 115–118. 28117445 10.1038/nature21056 PMC8382232

[46] Ehteshami Bejnordi B , Veta M , Johannes van Diest P , van Ginneken B , Karssemeijer N , Litjens G , van der Laak JAWM , the CAMELYON16 Consortium , Hermsen M , Manson QF *et al*. (2017) Diagnostic assessment of deep learning algorithms for detection of lymph node metastases in women with breast cancer. JAMA 318, 2199–2210. 29234806 10.1001/jama.2017.14585 PMC5820737

[47] Zhou J & Troyanskaya OG (2015) Predicting effects of noncoding variants with deep learning‐based sequence model. Nat Methods 12, 931–934. 26301843 10.1038/nmeth.3547 PMC4768299

[48] Poirion OB , Chaudhary K , Huang S & Garmire LX (2020) DeepProg: an ensemble of deep‐learning and machine‐learning models for prognosis prediction using multi‐omics data. medRxiv 19010082. 10.1101/19010082 PMC8281595 34261540

[49] Esteva A , Robicquet A , Ramsundar B , Kuleshov V , DePristo M , Chou K , Cui C , Corrado G , Thrun S & Dean J (2019) A guide to deep learning in healthcare. Nat Med 25, 24–29. 30617335 10.1038/s41591-018-0316-z

[50] Norgeot B , Glicksberg BS & Butte AJ (2019) A call for deep‐learning healthcare. Nat Med 25, 14–15. 30617337 10.1038/s41591-018-0320-3

[51] Troyanskaya O , Trajanoski Z , Carpenter A , Thrun S , Razavian N & Oliver N (2020) Artificial intelligence and cancer. Nat Cancer 1, 149–152. 10.1038/s43018-020-0034-6 35122011

[52] Vasaikar SV , Straub P , Wang J & Zhang B (2018) LinkedOmics: analyzing multi‐omics data within and across 32 cancer types. Nucleic Acids Res 46, D956–D963. 29136207 10.1093/nar/gkx1090 PMC5753188

[53] Liu SH , Shen PC , Chen CY , Hsu AN , Cho YC , Lai YL , Chen FH , Li CY , Wang SC , Chen M *et al*. (2020) DriverDBv3: a multi‐omics database for cancer driver gene research. Nucleic Acids Res 48, D863–D870. 31701128 10.1093/nar/gkz964 PMC7145679

[54] Ott PA , Hu Z , Keskin DB , Shukla SA , Sun J , Bozym DJ , Zhang W , Luoma A , Giobbie‐Hurder A , Peter L *et al*. (2017) An immunogenic personal neoantigen vaccine for patients with melanoma. Nature 547, 217–221. 28678778 10.1038/nature22991 PMC5577644

[55] Hoof I , Peters B , Sidney J , Pedersen LE , Sette A , Lund O , Buus S & Nielsen M (2009) NetMHCpan, a method for MHC class I binding prediction beyond humans. Immunogenetics 61, 1–13. 19002680 10.1007/s00251-008-0341-z PMC3319061

[56] Chen B , Khodadoust MS , Olsson N , Wagar LE , Fast E , Liu CL , Muftuoglu Y , Sworder BJ , Diehn M , Levy R *et al*. (2019) Predicting HLA class II antigen presentation through integrated deep learning. Nat Biotechnol 37, 1332–1343. 31611695 10.1038/s41587-019-0280-2 PMC7075463

[57] Reynisson B , Alvarez B , Paul S , Peters B & Nielsen M (2020) NetMHCpan‐4.1 and NetMHCIIpan‐4.0: improved predictions of MHC antigen presentation by concurrent motif deconvolution and integration of MS MHC eluted ligand data. Nucleic Acids Res 48, W449–W454. 32406916 10.1093/nar/gkaa379 PMC7319546

[58] De Mattos‐Arruda L , Vazquez M , Finotello F , Lepore R , Porta E , Hundal J , Amengual‐Rigo P , Ng CKY , Valencia A , Carrillo J *et al*. (2020) Neoantigen prediction and computational perspectives towards clinical benefit: recommendations from the ESMO Precision Medicine Working Group. Ann Oncol 31, 978–990. 32610166 10.1016/j.annonc.2020.05.008 PMC7885309

[59] Jiang D , Liao J , Duan H , Wu Q , Owen G , Shu C , Chen L , He Y , Wu Z , He D *et al*. (2020) A machine learning‐based prognostic predictor for stage III colon cancer. Sci Rep 10, 1–9. 32587295 10.1038/s41598-020-67178-0 PMC7316723

[60] Kermany DS , Goldbaum M , Cai W , Valentim CCS , Liang H , Baxter SL , McKeown A , Yang G , Wu X , Yan F *et al*. (2018) Identifying medical diagnoses and treatable diseases by image‐based deep learning. Cell 172, 1122–1131.e9. 29474911 10.1016/j.cell.2018.02.010

[61] Yamada M , Saito Y , Imaoka H , Saiko M , Yamada S , Kondo H , Takamaru H , Sakamoto T , Sese J , Kuchiba A *et al*. (2019) Development of a real‐time endoscopic image diagnosis support system using deep learning technology in colonoscopy. Sci Rep 9, 14465. 31594962 10.1038/s41598-019-50567-5 PMC6783454

[62] Hollon TC , Pandian B , Adapa AR , Urias E , Save AV , Khalsa SSS , Eichberg DG , D’Amico RS , Farooq ZU , Lewis S *et al*. (2020) Near real‐time intraoperative brain tumor diagnosis using stimulated Raman histology and deep neural networks. Nat Med 26, 52–58. 31907460 10.1038/s41591-019-0715-9 PMC6960329

[63] Miranda‐Escalada A , Farré E & Krallinger M (2020) Named entity recognition, concept normalization and clinical coding: overview of the Cantemist track for cancer text mining in Spanish, corpus, guidelines, methods and results. In Proceedings of the Proceedings of the Iberian Languages Evaluation Forum (IberLEF 2020), CEUR Workshop Proceedings, pp. 303–323.

[64] Rajkomar A , Oren E , Chen K , Dai AM , Hajaj N , Hardt M , Liu PJ , Liu X , Marcus J , Sun M *et al*. (2018) Scalable and accurate deep learning with electronic health records. NPJ Digit Med 1, 18. 31304302 10.1038/s41746-018-0029-1 PMC6550175

[65] Bowton E , Field JR , Wang S , Schildcrout JS , Van Driest SL , Delaney JT , Cowan J , Weeke P , Mosley JD , Wells QS *et al*. (2014) Biobanks and electronic medical records: enabling cost‐effective research. Sci Transl Med 6, 234cm3. 10.1126/scitranslmed.3008604 PMC4226414 24786321

[66] Topol EJ (2019) A decade of digital medicine innovation. Sci Transl Med 11, eaaw7610. 31243153 10.1126/scitranslmed.aaw7610

[67] Jim HSL , Hoogland AI , Brownstein NC , Barata A , Dicker AP , Knoop H , Gonzalez BD , Perkins R , Rollison D , Gilbert SM *et al*. (2020) Innovations in research and clinical care using patient‐generated health data. CA A Cancer J Clin 70, 182–199. 10.3322/caac.21608 PMC7488179 32311776

[68] Willems SM , Abeln S , Feenstra KA , de Bree R , van der Poel EF , Baatenburg de Jong RJ , Heringa J & van den Brekel MWM (2019) The potential use of big data in oncology. Oral Oncol 98, 8–12. 31521885 10.1016/j.oraloncology.2019.09.003

[69] Liu J , Lichtenberg T , Hoadley KA , Poisson LM , Lazar AJ , Cherniack AD , Kovatich AJ , Benz CC , Levine DA , Lee AV *et al*. (2018) An integrated TCGA pan‐cancer clinical data resource to drive high‐quality survival outcome analytics. Cell 173, 400–416.e11. 29625055 10.1016/j.cell.2018.02.052 PMC6066282

[70] Yap TA , Aerts JG , Popat S & Fennell DA (2017) Novel insights into mesothelioma biology and implications for therapy. Nat Rev Cancer 17, 475–488. 28740119 10.1038/nrc.2017.42

[71] Kondola S , Manners D & Nowak AK (2016) Malignant pleural mesothelioma: an update on diagnosis and treatment options. Ther Adv Respir Dis 10, 275–288. 26873306 10.1177/1753465816628800 PMC5933604

[72] Greiner KA , Friedman DB , Adams SA , Gwede CK , Cupertino P , Engelman KK , Meade CD & Hébert JR (2014) Effective recruitment strategies and community‐based participatory research: community networks program centers’ recruitment in cancer prevention studies. Cancer Epidemiol Biomarkers Prev 23, 416–423. 24609851 10.1158/1055-9965.EPI-13-0760 PMC3971731

[73] Unger JM , Cook E , Tai E & Bleyer A (2016) The role of clinical trial participation in cancer research: barriers, evidence, and strategies. Am Soc Clin Oncol Educ Book 36, 185–198. 10.14694/EDBK_156686 PMC5495113 27249699

[74] Russell SJ & Norvig P (2010) E. Artificial Intelligence: A Modern Approach. Prentice Hall, Upper Saddle River, NJ. ISBN 9780136042594.

[75] Mohri M , Rostamizadeh A & Talwalkar A (2012) Foundations of Machine Learning. MIT Press, Cambridge, MA, ISBN 9780262018258.

[76] Bishop CM (2006) Pattern Recognition and Machine Learning. Springer, New York, NY. ISBN 9780387310732.

[77] Ng AY & Jordan MI (2002) On discriminative vs. generative classifiers: a comparison of logistic regression and naive Bayes. In Advances in Neural Information Processing Systems 14 ( Dietterich TG , Becker S & Ghahramani Z , eds), pp. 841–848.MIT Press, Cambridge, MA.

[78] Ching T , Himmelstein DS , Beaulieu‐Jones BK , Kalinin AA , Do BT , Way GP , Ferrero E , Agapow PM , Zietz M , Hoffman MM *et al*. (2018) Opportunities and obstacles for deep learning in biology and medicine. J R Soc Interface 15, 20170387. 29618526 10.1098/rsif.2017.0387 PMC5938574

[79] Vabalas A , Gowen E , Poliakoff E & Casson AJ (2019) Machine learning algorithm validation with a limited sample size. PLoS One 14, e0224365. 31697686 10.1371/journal.pone.0224365 PMC6837442

[80] Smid SC , McNeish D , Miočević M & van de Schoot R (2020) Bayesian versus frequentist estimation for structural equation models in small sample contexts: a systematic review. Struct Equ Modeling 27, 131–161.

[81] McNeish D (2016) On using Bayesian methods to address small sample problems. Struct Equ Modeling 23, 750–773.

[82] Zondervan‐Zwijnenburg M , Peeters M , Depaoli S & Van de Schoot R (2017) Where do priors come from? Applying guidelines to construct informative priors in small sample research. Res Hum Dev 14, 305–320.

[83] Wang Y , Yao Q , Kwok JT & Ni LM (2020) Generalizing from a few examples. ACM Comput Surv 53, 1–34.

[84] Xian Y , Lampert CH , Schiele B & Akata Z (2019) Zero‐shot learning‐a comprehensive evaluation of the good, the bad and the ugly. IEEE Trans Pattern Anal Mach Intell 41, 2251–2265. 30028691 10.1109/TPAMI.2018.2857768

[85] Zoph B , Yuret D , May J & Knight K (2016) Transfer learning for low‐resource neural machine translation. In Proceedings of the Proceedings of the 2016 Conference on Empirical Methods in Natural Language Processing, pp. 1568–1575.Association for Computational Linguistics, Austin, TX.

[86] Hospedales T , Antoniou A , Micaelli P & Storkey A (2020) Meta‐learning in neural networks: a survey. arXiv. 2004.05439 [cs.LG]. 10.1109/TPAMI.2021.3079209 33974543

[87] Zhuang F , Qi Z , Duan K , Xi D , Zhu Y , Zhu H , Xiong H & He Q (2021) A comprehensive survey on transfer learning. Proc IEEE 109, 43–76.

[88] Zhu Y , Brettin T , Evrard YA , Partin A , Xia F , Shukla M , Yoo H , Doroshow JH & Stevens RL (2020) Ensemble transfer learning for the prediction of anti‐cancer drug response. Sci Rep 10, 18040. 33093487 10.1038/s41598-020-74921-0 PMC7581765

[89] Vanschoren J (2019) Meta‐Learning. In Automated Machine Learning: Methods, Systems Challenges ( Hutter F , Kotthoff L & Vanschoren J , eds), pp. 35–61.Springer International Publishing, Cham. ISBN 9783030053185.

[90] Qiu YL , Zheng H , Devos A , Selby H & Gevaert O (2020) A meta‐learning approach for genomic survival analysis. Nat Commun 11, 187. 33311484 10.1038/s41467-020-20167-3 PMC7733508

[91] Oliver A , Odena A , Raffel C , Cubuk ED & Goodfellow IJ (2018) Realistic evaluation of deep semi‐supervised learning algorithms. In Proceedings of the Proceedings of the 32nd International Conference on Neural Information Processing Systems, pp. 3239–3250.Curran Associates Inc., Red Hook, NY.

[92] van Engelen JE & Hoos HH (2020) A survey on semi‐supervised learning. Mach Learn 109, 373–440.

[93] Castro DC , Walker I & Glocker B (2020) Causality matters in medical imaging. Nat Commun 11, 3673. 32699250 10.1038/s41467-020-17478-w PMC7376027

[94] Goodfellow IJ , Pouget‐Abadie J , Mirza M , Xu B , Warde‐Farley D , Ozair S , Courville A & Bengio Y (2014) Generative adversarial nets. In Proceedings of the Proceedings of the 27th International Conference on Neural Information Processing Systems ‐ Volume 2, pp. 2672–2680.MIT Press, Cambridge, MA.

[95] Kingma DP & Welling M (2019) An introduction to variational autoencoders. FNT in Machine Learning 12, 307–392.

[96] Vaswani A , Shazeer N , Parmar N , Uszkoreit J , Jones L , Gomez AN , Kaiser L & Polosukhin I (2017) Attention is all you need. arXiv. 1706.03762 [cs.CL].

[97] Alqahtani H , Kavakli‐Thorne M & Kumar G (2019) Applications of generative adversarial networks (GANs): an updated review. Arch Comput Methods Eng 9, 147.

[98] Shorten C & Khoshgoftaar TM (2019) A survey on image data augmentation for deep learning. J Big Data 6, 1106. 10.1186/s40537-021-00492-0 PMC8287113 34306963

[99] Fernandez A , Garcia S , Herrera F & Chawla NV (2018) SMOTE for learning from imbalanced data: progress and challenges, marking the 15‐year anniversary. J Artif Intell Res 61, 863–905.

[100] Goncalves A , Ray P , Soper B , Stevens J , Coyle L & Sales AP (2020) Generation and evaluation of synthetic patient data. BMC Med Res Methodol 20, 108. 32381039 10.1186/s12874-020-00977-1 PMC7204018

[101] Björnsson B , Borrebaeck C , Elander N , Gasslander T , Gawel DR , Gustafsson M , Jörnsten R , Lee EJ , Li X , Lilja S *et al*. (2019) Digital twins to personalize medicine. Genome Med 12, 4. 31892363 10.1186/s13073-019-0701-3 PMC6938608

[102] Hanahan D & Weinberg RA (2011) Hallmarks of cancer: the next generation. Cell 144, 646–674. 21376230 10.1016/j.cell.2011.02.013

[103] de Anda‐Jáuregui G & Hernández‐Lemus E (2020) Computational oncology in the multi‐omics era: state of the art. Front Oncol 10, 423. 32318338 10.3389/fonc.2020.00423 PMC7154096

[104] Tan A , Huang H , Zhang P & Li S (2019) Network‐based cancer precision medicine: a new emerging paradigm. Cancer Lett 458, 39–45. 31125640 10.1016/j.canlet.2019.05.015

[105] Filipp FV (2019) Opportunities for artificial intelligence in advancing precision medicine. Curr Genet Med Rep 7, 208–213. 31871830 10.1007/s40142-019-00177-4 PMC6927552

[106] Topol EJ (2019) High‐performance medicine: the convergence of human and artificial intelligence. Nat Med 25, 44–56. 30617339 10.1038/s41591-018-0300-7

[107] Larson DB , Harvey H , Rubin DL , Irani N , Tse JR & Langlotz CP (2020) Regulatory frameworks for development and evaluation of artificial intelligence‐based diagnostic imaging algorithms: summary and recommendations. J Am Coll Radiol S1546‐1440, 31020–6. 10.1016/j.jacr.2020.09.060 PMC7574690 33096088

